# Relationship Between Litter Weight Gain and Colostrum Fatty Acid Composition: Implications for Cross-Fostering?

**DOI:** 10.3390/ani16060957

**Published:** 2026-03-19

**Authors:** Stephan Rosengart, Michael Wendt, Florian Lohkamp, Hubert Henne, Anne Kathrin Appel, Lea-Sophie Trost, Jens Tetens, Imke Traulsen, Ansgar Deermann, Christian Visscher

**Affiliations:** 1 Institute for Animal Nutrition, University of Veterinary Medicine Hannover, Foundation, Bischofsholer Damm 15, D-30173 Hannover, Germany; 2 Clinic for Swine and Small Ruminants, Forensic Medicine and Ambulatory Service, University of Veterinary Medicine Hannover, Foundation, Bischofsholer Damm 15, D-30173 Hannover, Germany; 3 BHZP GmbH, An der Wassermühle 8, D-21368 Ellringen, Germany; 4 Department of Animal Sciences, Livestock Systems, University of Göttingen, Albrecht-Thaer-Weg 3, D-37075 Göttingen, Germany; 5 Department of Animal Sciences, Functional Breeding, University of Göttingen, Burckhardtweg 2, D-37077 Göttingen, Germany; 6 Center of Integrated Breeding Research, University of Göttingen, Carl-Sprengel-Weg 1, D-37075 Göttingen, Germany; 7 Institute of Animal Breeding and Husbandry, Kiel University (CAU), Hermann-Rodewald-Straße 6, D-24116 Kiel, Germany; 8 EVH Select GmbH, An der Feuerwache 14, D-49716 Meppen, Germany

**Keywords:** colostrum composition, fatty acids, litter performance, piglets, sow

## Abstract

Colostrum is the primary source of nutrients for newborn piglets, significantly influencing their growth, health and survival. This study examines the relationship between sow litter weight gain and the fatty acid composition of colostrum, including crude protein, lactose, calculated gross energy and total solids. The n-6:n-3 fatty acid ratio was higher in low-performing sows (*p* < 0.05), suggesting that a lower ratio, associated with higher n-3 fatty acid content, may positively impact litter weight gain. Specific fatty acids such as myristic acid (C14:0), pentadecanoic acid (C15:0), palmitic acid (C16:0), palmitoleic acid (C16:1), margaric acid (C17:0), elaidic acid (C18:1n9t), linoleic acid (C18:2n6c), α-linolenic acid (C18:3n3), behenic acid (C22:0), docosadienoic acid (C22:2) and eicosapentaenoic acid (C20:5n3) were also found in lower concentrations in the colostrum of low-performing sows (*p* < 0.05). The study suggests that the content of n-3 fatty acids and perhaps the n-6:n-3 ratio of the colostrum and the content of some special fatty acids could serve as a selection criterion for improving litter performance in the future.

## Simple Summary

Newborn piglets depend on their mother’s first milk, called colostrum, for energy and protection against disease. Taking in enough high-quality colostrum shortly after birth is essential for their survival and growth. This study investigates the relationship between a sow’s litter weight gain and the composition of her colostrum, specifically focusing on many fatty acids, lactose, calculated gross energy and crude protein. Sows with lower litter weight gain produced colostrum with lower concentrations of myristic acid (C14:0), pentadecanoic acid (C15:0), palmitic acid (C16:0), palmitoleic acid (C16:1), margaric acid (C17:0), elaidic acid (C18:1n9t), linoleic acid (C18:2n6c), α-linolenic acid (C18:3n3), behenic acid (C22:0), docosadienoic acid (C22:2) and eicosapentaenoic acid (C20:5n3). In addition, these low-performing sows showed a lower content of n-3 fatty acids and a higher n-6:n-3 ratio in their colostrum. Thus, the study suggests that a low n-6:n-3 ratio and the levels of specific fatty acids in colostrum could be valuable indicators for selecting sows with improved litter performance. The study proposes that, if rapid testing methods for these components were available, this approach could become a useful tool for optimising cross-fostering strategies and enhancing overall piglet health and growth. In addition, heavier sows at weaning had litters with lower weight gain.

## 1. Introduction

Colostrum is the most important nutrient source for newborn piglets [1,2,3]. It provides essential nutrients such as carbohydrates, proteins and fats. Early energy supply by colostrum is particularly important because newborn piglets have only limited glycogen reserves [3,4]. Additionally, piglets with low birth weight have even lower glycogen reserves compared to those with higher birth weights [3,5].

In recent years, the number of live-born piglets per sow per year has increased, mainly due to genetic progress [6,7,8]. As a result, there are now both proportionally and absolutely more piglets per litter with low birth weights, as larger litters lead to greater variation in birth weights [9,10,11]. However, colostrum production has not increased proportionally [12]. This means that less colostrum is available per piglet than in the past [13,14], while at the same time, more piglets are born with low glycogen reserves [3,5]. Ensuring that every piglet receives a sufficient amount of colostrum and milk has therefore become more challenging than before.

It is clear that only healthy sows can achieve optimal colostrum and milk production [15,16,17]. Even then, providing a sufficient amount of colostrum for all piglets remains a challenge. In this context, it is important to maximise colostrum quantity and also to ensure the highest possible quality. Nevertheless, assessing colostrum quality is significantly more difficult than measuring its quantity. In any case, evaluating colostrum quality requires analysing its components. A proper interpretation of the analysis results requires an understanding of the physiological effects of individual colostrum components.

Colostrum is an irreplaceable carrier of maternal antibodies and functional oligosaccharides, which help develop the piglet’s immune system [18,19]. High immunoglobulin levels appear to be an important quality criterion, and there is a correlation between the amount of colostrum ingested and the pre-weaning mortality rate of piglets [20]. The fat content and fatty acid composition of sow colostrum can be influenced by diet [21,22,23,24,25]. While the fatty acid profile and content of total fat in colostrum can be altered by sow nutrition [21,22,23,26,27], the effects of different fatty acid compositions contained in the colostrum on the piglets have not yet been thoroughly investigated. It is already known that n-3 polyunsaturated fatty acids, such as docosahexaenoic acid (C22:6n3), trigger anti-allergic and anti-inflammatory immune responses. In contrast, n-6 polyunsaturated fatty acids, such as arachidonic acid (C20:4n6), promote pro-inflammatory immune responses [28,29,30,31]. A high proportion of linoleic acid and α-linolenic acid in the total fat content of colostrum was shown to have a positive effect on the daily weight gain of piglet litters [26]. Furthermore, the lactose content appears to correlate negatively with the IgG content, and the protein content appears to correlate positively with the IgG content [32,33,34]. In addition, the fatty acid profile in pig feed has an influence on the immune system of pigs [35].

The aim of this study was to examine whether colostrum from sows with high litter weight gain differs from that of sows with comparatively low litter weight gain. We hypothesise that there are many associations between performance data and colostrum composition in this regard. The study compares parameters including total fat content, individual fatty acid composition, dry matter content, lactose content, calculated gross energy and crude protein content.

## 2. Materials and Methods

### 2.1. Animals, Housing and Feeding

The investigation was conducted on a farrow-to-feeder pig farm located in Lower Saxony, Northern Germany, spanning the period from August 2019 to February 2021. All sows were housed under uniform environmental conditions, with room temperature regulated by an automated climate control system. The farm kept approximately 500 hybrid sows (db.Viktoria, BHZP Large White × BHZP Landrace) from the German Federal Hybrid Breeding Programme (BHZP GmbH, Ellringen, Germany), and batch farrowing was implemented at two-week intervals. The facility comprised four farrowing units, each equipped with 24 farrowing pens (ProDromi, Vereijken Hooijer B.V., Ede, The Netherlands), arranged with 12 pens on either side of a central working aisle. Fresh air was provided by automated, negative-pressure ventilation via central exhaust fans and a porous ceiling system.

On day 109 of gestation—seven days prior to the expected farrowing date (designated as day −7)—the sows were transferred to the farrowing pens, where they remained in farrowing crates until weaning. From day −7 to day −3, the sows received a gestation diet administered twice daily. Between day −2 and day 0, feeding was reduced to once daily with a transitional mix of gestation and lactation diets. From day 1 to day 2, sows were again fed twice daily, and from day 3 until weaning, they received a lactation diet three times per day. The nutrient composition of the sow diets is presented in detail in Table 1 and was determined using the analytical methods of the Association of German Agricultural Analytical and Research Institutes (VDLUFA) [36]. Feed formulation followed the guidelines of the Society for Nutritional Physiology (GfE) [37]. During gestation, every sow received an individual amount of feed.

The farrowing period extended from day −3 to day 1. Every piglet was weighed and individually marked within 24 h postpartum, with a second weighing of every piglet conducted shortly before weaning. Sows were also weighed upon entry into the farrowing unit and again at weaning. All animals had continuous access to tap water.

### 2.2. Sampling

As part of the study, a single colostrum sample was collected from each of the 280 sows across 41 farrowing groups, with 5 to 10 samples taken per group. The sows were selected at random. Sampling was performed close to the time of farrowing and within a maximum of six hours after the onset of parturition. Colostrum was collected from sows after parturition of three to four piglets, with at least one piglet still being wet at the time of sampling. Prior to sample collection, the mammary glands were dry-cleaned and massaged, followed by manual milking of all functional teats to obtain 10 to 20 mL of colostrum per sow (sample tubes: Schraubröhre, 30 mL, 84 × 30 mm, Sarstedt AG & Co. KG, Nümbrecht, Germany). The samples were pooled from multiple teats. Immediately after collection, samples were individually labelled with the sow number and date, and immediately frozen at −20 °C [24,27,38]. None of the sampled sows had received antibiotic treatment within the seven days preceding sampling. Furthermore, no sow had undergone medical induction of parturition. Parity information was obtained via the herd management software db.Planer (version 1906, BHZP GmbH).

### 2.3. Analysis of Colostrum

The measurement of the content of fatty acids in the colostrum was carried out in accordance with modified established methods [39]. A 100 mg colostrum sample was poured into a narrow glass tube. An internal standard consisting of 2 mL methanol-hexane-tridecanoic acid (C13) mixture was added to each colostrum sample. Acetyl chloride (200 µL) was added, and the mixture was heated for one hour at 100 °C. Afterwards, 5 mL of potassium carbonate chloride solution was added. Fatty acids were chromatographed as methyl esters on a 100 m fused silica column with an internal diameter of 0.25 mm (Restek GmbH, Bad Homburg, Germany). The column was wall-coated with 0.20 mm SP-2330. Analysis was performed on a gas chromatograph Trace 1300 (Thermo Fisher Scientific GmbH, Dreieich, Germany) equipped with a flame ionisation detector. Nitrogen was used as a carrier gas. The split ratio was 67:1. The injection port temperature was 280 °C, and the temperature of the detector was 260 °C. The column temperature was kept at 50 °C for 2 min and, in a stepwise (30 °C/min) fashion, reached a plateau of 120 °C. This temperature was maintained for 1 min. Afterwards, the temperature was increased stepwise at a rate of 3.5 °C per min until reaching a plateau of 240 °C, which was maintained for 12 min. External standard for identifying individual fatty acids was FAME Mix, C4-C24, 18919-1AMP (Merk KGaA, Darmstadt, Germany). The total fatty acid content was calculated as the sum of the individual fatty acid concentrations.

Crude protein concentration was determined as nitrogen using the Dumas method, and the crude protein content was calculated according to VDLUFA guidelines. The sample (500 µL) was weighed and afterwards combusted at 1140 °C in a carrier gas stream with the addition of oxygen with a rapid Max N Exceed (Elementar Analysensysteme GmbH, Langenselbold, Germany). After reducing the formed nitrogen oxides to molecular nitrogen and removing other combustion products by selective absorption, the molecular nitrogen was detected by the machine using a thermal conductivity detector (Elementar Analysensysteme GmbH, Langenselbold, Germany). Data evaluation was carried out using the instrument-specific rapid Max N Exceed software (version 1.1.16, Elementar Analysensysteme GmbH, Langenselbold, Germany). All analyses were performed in duplicate. Energy content was calculated by using the following formula from the literature [40]:

GE (MJ/kg) = (0.057 CP% + 0.092 fat% + 0.0395 lactose%) × 4.1868.

### 2.4. Compared Parameters

In total, 280 colostrum samples were analysed. The low-performing group consisted of 140 samples collected from sows exhibiting the lowest litter weight gain, whereas the high-performing group comprised 140 samples obtained from sows with the highest litter weight gain. Litter weight gain was calculated by multiplying the average daily weight gain of a suckling piglet by the number of weaned piglets and by 18 days of lactation. The average time from birth to the second weighing was 18 days. In addition, the average daily weight gain of a suckling piglet was calculated individually for every litter. Subsequently, the 140 colostrum samples from the high-performing group were compared with the 140 colostrum samples from the low-performing group to maximise the contrast between groups and identify potential biomarkers of performance.

### 2.5. Statistical Analysis

The statistical analysis was conducted using SAS Enterprise Guide (version 7.1, SAS Institute Inc., Cary, NC, USA). For all parameters, mean values and standard deviations were calculated. Differences between performance groups were assessed using a one-way analysis of variance (ANOVA). For multiple pairwise means comparison between the two groups, the Ryan–Einot–Gabriel–Welsch multiple-range test (REGWQ) was used. A *p*-value of less than 0.05 was considered statistically significant.

## 3. Results

### 3.1. Colostrum Composition

The three fatty acids with the highest concentration were palmitic acid (C16:0), oleic acid (C18:1n9c) and linoleic acid (C18:2n6c) (Table 2). Their total concentration in the colostrum of sows with low litter weight gain was 27.5 g/kg ± 11.4, while in sows with high litter weight gain, it was 30.2 g/kg ± 13.8 (*p* > 0.05). Their proportion of total fatty acids in the respective groups was 83.2% ± 0.8 (low-performing sows) and 83.0% ± 1.0 (high-performing sows).

The content of some fatty acids was significantly lower in the colostrum of the low- performing sows. This applied to the fatty acids myristic acid (C14:0), pentadecanoic acid (C15:0), palmitic acid (C16:0), palmitoleic acid (C16:1), margaric acid (C17:0), elaidic acid (C18:1n9t), α-linolenic acid (C18:3n3), behenic acid (C22:0), docosadienoic acid (C22:2) and eicosapentaenoic acid (C20:5n3). Regarding the content of n-3 fatty acids and the n-6:n-3 ratio, the colostrum from high-performing sows contained more n-3 fatty acids than the colostrum from low-performing sows. Thus, the n-6:n-3 ratio was lower in colostrum of high-performing sows (*p* < 0.05, Table 2).

### 3.2. Sows’ Data

In the low-performing group, the mean litter weight gain was 35.38 kg ± 7.18 and in the high-performing group, 49.37 kg ± 4.95 within the first 18 days of life (*p* < 0.05, Table 3). Furthermore, the body weight (BW) of the high-performing sows at weaning was significantly lower than that of the low-performing sows (*p* < 0.05, Table 3). However, there were no significant differences between the two groups with regard to BW when entering the farrowing room and with regard to parity (*p* > 0.05, Table 3).

## 4. Discussion

The most important nutrient source for newborn piglets is colostrum [1,2,3]. Quality of colostrum is crucial for piglet growth, health and survival [41,42,43,44]. Its production is influenced by various factors [41,43,45], including sow and litter characteristics, nutrition, hormonal and metabolic status, as well as environmental conditions, either individually or in combination [46]. This study examines the relationship between a sow’s litter weight gain and the concentration of its individual fatty acids, crude protein, lactose, calculated gross energy and total solids in colostrum.

### 4.1. Content of n-3 Fatty Acids and Ratio of n-6:n-3 Fatty Acids

The content of n-3 fatty acids and the n-6:n-3 ratio were higher in colostrum of low-performing sows (*p* < 0.05). You et al. [47] reported a higher daily weight gain of piglets until weaning when the lactation diet of the sows was supplemented with 1 g sodium butyrate per kg diet, 7.75 g medium-chain fatty acids (MCFA) per kg diet and 68.2 g n-3 polyunsaturated fatty acids (PUFA) per kg diet. All three components seemed to have a synergistic effect. The addition of n-3 fatty acids appeared to have the greatest impact. Luo et al. [48] and Llauradó-Calero et al. [49] reported a significantly higher daily weight gain in piglets and a positive effect on the immune system of sows and piglets fed with a higher content of n-3 fatty acids through fish oil supplementation. In another study, alpha-gylcerol monolaurate was added to the gestation and lactation diet [24]. This did not result in any changes in the colostrum. In milk, the proportion of n-3 fatty acids increased, even though alpha-glycerol monolaurate does not contain any n-3 fatty acids. Improved absorption processes in the intestine through alpha-glycerol monolaurate were discussed as possible causes. In addition, changes in milk composition had a positive effect on piglet growth [24]. Interestingly, the addition of 500 mg of alpha-glycerol monolaurate per kg of feed in the aforementioned study had the same effect regarding the piglets’ weight gain as the addition of 1000 or 2000 mg [24]. The content of lauric acid in the colostrum increased proportionally to the amount of alpha-glycerol monolaurate added. Overall, both our data and the studies by Li et al., 2023 [24] and You et al., 2023 [47] suggest that high n-3 fatty acid levels in colostrum and milk have a positive effect on piglet weight gain. This is likely to be true up to a threshold value beyond which no further benefit for the piglets can be measured. However, exceeding this limit has no negative effects for the piglets. In addition, lauric acid is described in the literature as a fatty acid with antimicrobial activity [50]. Nevertheless, the absolute ratios observed in our study differ from those reported in the literature (low-performing: 15.33 vs. 10.29; low-performing: 14.86 vs. 9.24) [24]. Overall, the findings of this study suggest that especially the content of n-3 fatty acids and perhaps the n-6:n-3 ratio are important indicators of colostrum quality. However, measuring it is currently quite complex. If rapid tests are developed in the future, the situation would change significantly. Management strategies such as selection decisions, feeding strategies and cross-fostering could be optimised based on the n-6:n-3 ratio. Nevertheless, further research is needed to confirm this conclusively.

### 4.2. Composition of Some Individual Fatty Acids

Low-performing sows showed a significantly lower content of myristic acid (C14:0) in their colostrum (*p* < 0.05). While direct evidence in pigs is lacking, studies in other species suggest that myristic acid (C14:0) has an anti-inflammatory activity on human macrophages in vitro and on mice with induced chronic skin inflammation [51]. Nothing can be found in the literature about the effect of myristic acid in pigs. However, pigs serve as an excellent model for human infectious diseases, biomedical research and toxicological testing due to their similar physiological processes [52,53]. Therefore, it may be hypothesised that the higher content of myristic acid in the colostrum of the high-performing sows is an explanation for the better growth of the piglets because of a potentially anti-inflammatory effect in piglets. In addition, myristic acid is a common component of cell membrane phospholipids and influences the membrane fluidity of red blood cells in monks [54]. Thus, it may influence the membrane fluidity of piglets, too.

Low-performing sows showed a significantly lower content of pentadecanoic acid (C15:0) in their colostrum (*p* < 0.05). While direct evidence in pigs is lacking, studies in humans showed a negative correlation between the incidence of type 2 diabetes (T2D) and metabolic dysfunction-associated steatotic liver disease (MASLD) and plasma concentrations of pentadecanoic acid (C15:0) [55,56]. In mice, dietary supplementation of pentadecanoic acid (C15:0) reduced aspartate transaminase (AST), alanine transaminase (ALT) and pro-inflammatory cytokines (TNF-a and IL-6). The examined mice were fed a choline-deficient high-fat diet [57]. Assuming similar effects in pigs, a lower level of pro-inflammatory cytokines may explain the improved performance of piglets that received colostrum with a higher concentration of pentadecanoic acid (C15:0).

Palmitic acid (C16:0) had the second-highest concentration in the colostrum of both groups, following oleic acid (C18:1n9c). In addition, in the colostrum of low-performing sows, the palmitic acid (C16:0) content was significantly lower compared to that of high-performing sows (*p* < 0.05). A previous study showed a relationship between the palmitic acid (C16:0) content in colostrum and milk and the BW of suckling piglets [58]. However, piglets that consumed colostrum and milk with low palmitic acid levels had a higher BW than those that consumed colostrum and milk with high palmitic acid levels. That study’s findings therefore appear to contradict the results of our current study. However, it is important to note that the mentioned study did not clearly distinguish between colostrum and milk, which makes its conclusion less precise; Skrzypczak, Waśkiewicz, Beszterda, Goliński, Szulc, Buczyński and Babicz [58] summarised the values of both colostrum and milk.

Low-performing sows showed a significantly lower content of palmitoleic acid (C16:1) in their colostrum (*p* < 0.05). As palmitoleic acid (C16:1) has anti-inflammatory potential in human endothelial cells [59], it may have positive effects on the development of piglets.

Low-performing sows showed a significantly lower content of margaric acid (C17:0) in their colostrum (*p* < 0.05). In mice, margaric acid (C17:0) can be endogenously synthesised via alpha-oxidation from straight-chain precursors [60]. In pigs, this fatty acid does not appear to be of major importance.

Low-performing sows showed a significantly lower content of elaidic acid (C18:1n9t) in their colostrum (*p* < 0.05). However, there is no information in the literature about the effects in pigs.

Low-performing sows showed a significantly lower content of α-linolenic acid (C18:3n3) in their colostrum (*p* < 0.05). The content of linoleic acid (C18:2n6c) tended to be lower in colostrum of low-performing sows (*p* < 0.1). Similar results were reported by Holen, Woodworth, Tokach, Goodband, DeRouchey, Gebhardt, DeDecker and Martinez [26] showing a higher daily weight gain in piglet litters when colostrum had significantly higher proportions of linoleic acid (C18:2n6c) and α-linolenic acid (C18:3n3).

The content of behenic acid (C22:0) was significantly lower in the colostrum of low-performing sows (*p* < 0.05). Behenic acid belongs to the long-chain fatty acids and its content increases in the milk of dairy cows if there is a negative energy balance (NEB) in the lactating animal [61]. For sows, this has not yet been demonstrated for behenic acid. However, there is an older study that was able to show this effect for other long-chain fatty acids (oleic acid (C18:1n-9) and vaccenic acid (C18:1n-7)) [62]. The sows in our study were restrictively fed during the peripartal period. Therefore, sows bearing a large number of piglets may have entered an NEB during the peripartal period.

The content of docosadienoic acid (C22:2) was significantly lower in the colostrum of low-performing sows compared to that of high-performing sows (*p* < 0.05). A previous study added 5% glycerol to the lactation diet of sows and compared milk composition and performance, including the weaning weight of piglets, with sows that were fed a lactation diet without glycerol supplementation [63]. The piglets’ weaning weight did not change, but the content of docosadienoic acid (C22:2) in sows’ milk increased in the glycerol-supplemented group. This means that this fatty acid is transferred from the feed into the milk, but does not have a performance-enhancing effect on the piglets.

Eicosapentaenoic acid (C20:5n3) was significantly lower in the colostrum of low-performing sows compared to that of high-performing sows (*p* < 0.05). Eicosapentaenoic acid (C20:5n3) is part of the n-3 fatty acid group and is abundant in fish oil [64]. This fatty acid has been shown to inhibit various inflammatory responses in humans, including leucocyte chemotaxis, adhesion molecule expression and leucocyte-endothelial adhesive interactions [64]. An increased intake of n-3 fatty acids in the diet of lactating sows has been reported to have a positive effect on the immune system of piglets [48] and their social activities [65]. Nonetheless, the explicit content of eicosapentaenoic acid (C20:5n3) was not examined in the two previously mentioned studies [48,65]. Clouard, Souza, Gerrits, Hovenier, Lammers and Bolhuis [65] attributed the positive effect to the high content of docosahexaenoic acid (C22:6n3). Regarding the performing groups, the study presented here found no significant differences in the levels of docosahexaenoic acid (C22:6n3). Overall, it should be noted that we looked for correlations between the content of individual fatty acids in colostrum and litter weight gain but found none.

### 4.3. Sows’ Data

The BW of sows at weaning was significantly higher in low-performing sows than in high-performing sows (*p* < 0.05). In addition, low-performing sows tended to lose less weight compared with the high-performing group (*p* = 0.06). In sows that mobilise more body reserves (lose more weight), there may be proportionately greater partitioning of nutrients towards milk production, benefiting piglet growth, but this may have implications for subsequent reproductive performance. The mean parity of sows in the low-performing and high-performing groups was similar (4.72 vs. 4.44). Ajay et al. [66] confirmed that thin sows have higher litter weight gain than fat sows. However, the differentiation of the sows in their study into low, moderate and high body condition animals already took place when they were moved into the farrowing pen. In all three groups, approximately the same BW per animal was metabolised during lactation. Therefore, until weaning, the high body condition group remained the high body condition group, the moderate body condition group remained the moderate body condition group and the low body condition group remained the low body condition group (Ajay et al., 2023) [66]. It can be added that in our study the low-performing sows tended to be heavier than the high-performing sows at the time of entering the farrowing room (*p* < 0.1). At weaning, the difference was significant (*p* < 0.05). Therefore, in our study, the performance difference between the low-performing and high-performing groups can also be accounted for by BW loss. During gestation, every sow received an individual amount of feed. Therefore, none of the sows were too fat while farrowing.

## 5. Conclusions

The present study investigated the relationship between sow litter weight gain and colostrum composition, focusing on fatty acid profiles, crude protein and total solids. The results demonstrated that low-performing sows, characterised by lower litter weight gain, exhibited significantly lower levels of myristic acid (C14:0), pentadecanoic acid (C15:0), palmitic acid (C16:0), palmitoleic acid (C16:1), margaric acid (C17:0), elaidic acid (C18:1n9t), linoleic acid (C18:2n6c), α-linolenic acid (C18:3n3), behenic acid (C22:0), docosadienoic acid (C22:2) and eicosapentaenoic acid (C20:5n3) in their colostrum (*p* < 0.05), potentially impacting piglet growth and immune function. Furthermore, the n-6:n-3 fatty acid ratio was higher in colostrum of low-performing sows, suggesting that a lower ratio, indicative of higher n-3 fatty acid content, may positively influence litter weight gain. Overall, the findings suggest that colostrum fatty acid composition, particularly the content of n-3 fatty acids and perhaps the n-6:n-3 fatty acid ratio, plays a critical role in piglet growth and survival. Future research should explore the potential of the n-6:n-3 ratio and the aforementioned fatty acids as selection criteria for improving cross-fostering and litter performance. The approach could become practical if rapid tests for the examined colostrum components could be developed in the future. Further studies should investigate whether supplementation with specific fatty acids during late gestation can improve colostrum quality and subsequent litter performance.

## Figures and Tables

**Table 1:** Nutrient contents in the gestation and lactation diet (pelleted complete feed) in accordance with the analysis (g/kg as fed).

Item	Gestation Diet	Lactation Diet
Total solids	887 ± 7.5	889 ± 6.9
Crude protein	142 ± 3.4	163 ± 10.1
Crude fat	30.0 ± 1.5	36.0 ± 4.0
Crude fibre	72.0 ± 5.8	48.0 ± 3.7
Crude ash	53.0 ± 1.4	52.0 ± 3.0
Calcium	7.20 ± 1.0	8.10 ± 1.0
Phosphorus	4.80 ± 0.4	6.00 ± 0.2
Energy (MJ ME/kg)	11.7 ± 0.3	13.0 ± 0.3
Lauric acid (C12:0)	0.09 ± 0	<0.05
Myristic acid (C14:0)	0.08 ± 0.02	0.05 ± 0.01
Pentadecanoic acid (C15:0)	<0.05	<0.05
Palmitic acid (C16:0)	5.99 ± 0.18	6.06 ± 0.34
Palmitoleic acid (C16:1)	0.06 ± 0.01	0.06 ± 0.01
Margaric acid (C17:0)	<0.05	<0.05
Stearic acid (C18:0)	0.50 ± 0.01	0.70 ± 0.04
Elaidic acid (C18:1n9t)	<0.05	<0.05
Oleic acid (C18:1n9c)	4.61 ± 0.38	4.57 ± 0.36
Linoleic acid (C18:2n6c)	15.0 ± 0.42	18.5 ± 1.08
Arachidic acid (C20:0)	0.07 ± 0	0.07 ± 0.01
γ-Linolenic acid (C18:3n6)	<0.05	<0.05
Gondoic acid (C20:1)	0.17 ± 0.01	0.16 ± 0.01
α-linolenic acid (C18:3n3)	1.21 ± 1.2	1.82 ± 1.13
Eicosadienoic acid (C20:2)	0.05 ± 0	0.05 ± 0.002
Behenic acid (C22:0)	0.09 ± 0.002	0.09 ± 0.004
Dihomo-γ-linolenic acid (C20:3n6)	<0.05	<0.05
Erucic acid (C22:1n9)	<0.05	<0.05
Eicosatrienoic acid (C20:3n3)	<0.05	<0.05
Arachidonic acid (C20:4n6)	<0.05	<0.05
Docosadienoic acid (C22:2)	<0.05	<0.05
Lignoceric acid (C24:0)	0.10 ± 0.02	0.08 ± 0.01
Eicosapentaenoic acid (C20:5n3)	<0.05	<0.05
Nervonic acid (C24:1)	0.06 ± 0.002	0.05
Docosahexaenoic acid (C22:6n3)	<0.05	<0.05
Total fatty acids	28.0 ± 0.53	32.2 ± 1.91
n6:n3 ratio	12.5 ± 0.36	10.2 ± 0.48

**Table 2:** Comparison of contents of different fatty acids, crude protein, lactose, gross energy and total solids in colostrum of low-performing and high-performing sows. Data are shown in g/kg of original substance.

Item	Low-Performing	High-Performing	*p*-Value
	(N = 140)	(N = 140)	
Myristic acid (C14:0)	0.527 ^b^ ± 0.208	0.594 ^a^ ± 0.274	0.022
Pentadecanoic acid (C15:0)	0.037 ^b^ ± 0.034	0.047 ^a^ ± 0.041	0.025
Palmitic acid (C16:0)	8.925 ^b^ ± 3.701	9.911 ^a^ ± 4.490	0.046
Palmitoleic acid (C16:1)	1.033 ^b^ ± 0.464	1.173 ^a^ ± 0.617	0.033
Margaric acid (C17:0)	0.117 ^b^ ± 0.051	0.135 ^a^ ± 0.074	0.018
Stearic acid (C18:0)	2.229 ± 1.024	2.439 ± 1.128	0.105
Elaidic acid (C18:1n9t)	0.052 ^b^ ± 0.036	0.064 ^a^ ± 0.051	0.028
Oleic acid (C18:1n9c)	11.65 ± 5.170	12.65 ± 5.932	0.134
Linoleic acid (C18:2n6c)	6.931 ^†^ ± 0	7.599 ^†^ ± 0.007	0.091
Arachidic acid (C20:0)	0.046 ^†^ ± 0.033	0.054 ^†^ ± 0.044	0.096
γ-Linolenic acid (C18:3n6)	0.102 ± 0.056	0.106 ± 0.058	0.616
Gondoic acid (C20:1)	0.107 ± 0.052	0.114 ± 0.060	0.301
α-linolenic acid (C18:3n3)	0.457 ^b^ ± 0.181	0.517 ^a^ ± 0.257	0.025
Eicosadienoic acid (C20:2)	0.201 ± 0.082	0.214 ± 0.094	0.200
Behenic acid (C22:0)	0.013 ^b^ ± 0.024	0.025 ^a^ ± 0.034	0.001
Dihomo-γ-linolenic acid (C20:3n6)	0.093 ± 0.045	0.100 ± 0.054	0.263
Erucic acid (C22:1n9)	0 ^†^ ± 0	0.002 ^†^ ± 0.011	0.063
Eicosatrienoic acid (C20:3n3)	0.027 ± 0.031	0.033 ± 0.033	0.176
Arachidonic acid (C20:4n6)	0.391 ± 0.164	0.410 ± 0.185	0.373
Docosadienoic acid (C22:2)	0.006 ^b^ ± 0.016	0.011 ^a^ ± 0.023	0.027
Lignoceric acid (C24:0)	0.071 ± 0.029	0.072 ± 0.041	0.770
Eicosapentaenoic acid (C20:5n3)	0.007 ^b^ ± 0.019	0.013 ^a^ ± 0.024	0.019
Nervonic acid (C24:1)	0.041 ± 0.031	0.046 ± 0.034	0.189
Docosahexaenoic acid (C22:6n3)	0.011 ± 0.024	0.015 ± 0.029	0.187
Total fatty acids	33.08 ^†^ ± 13.7	36.35 ^†^ ± 16.6	0.074
Crude protein	144.2 ± 29.2	145.0 ± 33.7	0.836
Lactose	29.8 ± 5.92	28.2 ± 5.39	0.122
Gross Energy (MJ/kg) ^1^	5.23 ± 1.03	5.40 ± 1.07	0.367
Total solids	217.8 ± 31.5	222.0 ± 36.4	0.309
n-3	0.50 ^b^ ± 0.23	0.58 ^a^ ± 0.32	0.023
n-6	7.52 ± 3.07	8.21 ± 3.96	0.101
n-6:n-3 ratio	15.33 ^a^ ± 1.53	14.86 ^b^ ± 1.76	0.018

^1^ Gross energy was calculated using the equation from the literature [40]; ^a,b^ Values within a row with different superscripts differ significantly at *p* < 0.05. ^†^ Values within a row with this superscript tended to be different with *p* < 0.1 and *p* ≥ 0.05. Mean comparisons were analysed using the Ryan–Einot–Gabriel–Welsch multiple-range test (REGWQ).

**Table 3:** Comparison of sow’s body weight and parity between low-performing sows and high-performing sows from the same farrowing farm.

Item	Low-Performing	High-Performing	*p*-Value
	(N = 140)	(N = 140)	
LWG	35.38 ^b^ ± 7.18	49.37 ^a^ ± 4.95	<0.0001
NWP	11.36 ^b^ ± 1.89	12.74 ^a^ ± 1.40	<0.0001
SW1	279.6 ± 34.8	271.6 ± 35.9	0.059
SW2	235.5 ^a^ ± 43.8	225.0 ^b^ ± 36.8	0.031
SWD	−41.66 ± 22.0	−46.61 ± 22.2	0.063
Parity	4.72 ± 3.08	4.44 ± 2.54	0.398

LWG in kg = litter weight gain, NWP = number of weaned piglets, SW1 in kg = sow weight when entering the farrowing room, SW2 in kg = sow weight at weaning, SWD in kg = difference between SW1 and SW2. ^a,b^ = values within a row with different superscripts differ significantly at *p* < 0.05. Mean comparisons were analysed using the Ryan–Einot–Gabriel–Welsch multiple-range test (REGWQ).

## Data Availability

The data presented in this study are available in this manuscript. Further inquiries can be directed to the corresponding author.
